# Decitabine bioproduction using a biocatalyst with improved stability by adding nanocomposites

**DOI:** 10.1186/s13568-020-01109-0

**Published:** 2020-09-29

**Authors:** Mariana B. Méndez, Jorge A. Trelles, Cintia W. Rivero

**Affiliations:** 1 Laboratory of Sustainable Biotechnology (LIBioS), National University of Quilmes, Roque Sáenz Peña 352, B1876BXD Bernal, Argentina; 2 National Scientific and Technical Research Council (CONICET), Godoy Cruz 2290, C1425FQB Caba, Argentina

**Keywords:** Biocatalysis, Nucleoside 2′-deoxyribosyltransferase, Bentonite, Immobilization

## Abstract

A novel IDA-*La*NDT derivative was able to reach the highest productivity in the biosynthesis of a well-known antitumoral agent called decitabine. However, the combination of two simple and inexpensive techniques such as ionic absorption and gel entrapment with the incorporation of a bionanocomposite such as bentonite significantly improved the stability of this biocatalyst. These modifications allowed the enhancement of storage stability (for at least 18 months), reusability (400 h of successive batches without significant loss of its initial activity), and thermal and solvent stability with respect to the non-entrapped derivative. Moreover, reaction conditions were optimized by increasing the solubility of 5-aza by dilution with dimethylsulfoxide. Therefore, a scale-up of the bioprocess was assayed using the developed biocatalyst, obtaining 221 mg/L·h of DAC. Finally, green parameters were calculated using the nanostabilized biocatalyst, whose results indicated that it was able to biosynthesize DAC by a smooth, cheap, and environmentally friendly methodology.
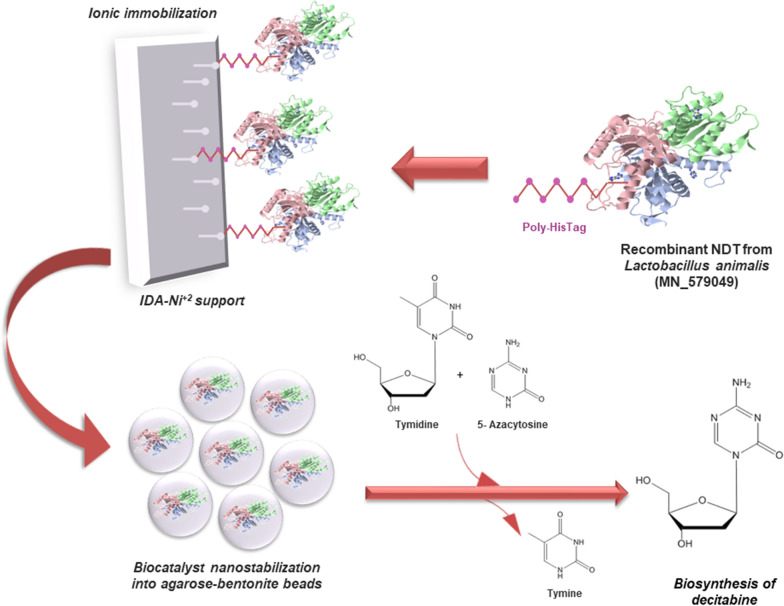

## Key points

- *L. animalis* NDT was successfully immobilized by ionic absorption and gel entrapment
- The enzymatic nanobiocatalyst with improved stability was able to produce decitabine
- Bioprocess scale-up allowed developing an eco-compatible technology

## Introduction

The nucleoside analog 5-aza-2′-deoxycytidine (decitabine, DAC) is a potent antileukemic agent, currently used in chemotherapy for the treatment of myelodysplastic syndromes (MDS), acute myelodysplastic leukemia (AML), chronic myelogenous leukemia (CML), which is nowadays in clinical trial for other human cancers and sickle cell anemia (Dinardo et al. 2019; Briot et al. 2017; Issa et al. 2004). Besides, there is ample evidence that numerous epigenetic mutations are directly linked with carcinogenesis, such as the commonly identified aberrant DNA methylation in AML disorder (Jabbour et al. 2008). Because these kinds of mutations are reversible, they comprise potential therapeutic targets by nucleoside analogs such as DAC, which has proven to have a high efficiency in AML treatment by involving the use of DNA methyltransferase (DNMT) inhibitors (Ganetsky 2012).

Biocatalysts (both isolated enzyme and whole-cell systems) are increasingly being used to assist in conventional synthetic routes as well as to create new routes of complex molecules of industrial interest, in order to reduce the cost and the environmental impact of the production of compounds of higher value. Under this perspective, biocatalysis advantages lie in the regio- and stereoselective properties of enzymes, which are able to react under mild reaction conditions (aqueous environment, physiological pH, ambient temperature/pressure) without the need to protect the existing functionality (Pollard and Woodley 2007). In this way, biocatalysis is presented today as an alternative green process able to revolutionize the conventional chemical synthesis.

On the other hand, nucleoside 2′-deoxyribosyltransferases (NDTs; EC 2.4.2.6) are enzymes highly used to catalyze nucleoside synthesis by the transfer of glycosyl residues to acceptor bases (Fresco-Taboada et al. 2013). These enzymes are classified into two classes depending on their substrate specificity: NDT type I (PDT), specific for purines, and NDT type II (NDT), which catalyzes the transfer between purines and/or pyrimidines (Lapponi et al. 2016). In the last years, NDTs have demonstrated their versatility in the biosynthesis of diverse nucleoside analogs commonly used as antiretroviral or antitumoral agents, as in the case of NDT from *Lactobacillus reuteri* (Fernández-Lucas et al. 2011, 2013, 2010), *Lactobacillus animalis* (Britos et al. 2016; Cappa et al. 2014; Méndez et al. 2018; Rivero et al. 2012), and *Lactobacillus helveticus* (Liang et al. 2010), among others.

However, the use of soluble enzymes in catalysis has many limitations because of the high cost of recombinant enzymes, low stability and the complicated downstream processing to recover the enzyme from the reaction media (Zucca et al. 2016). Enzyme immobilization emerges as an alternative to overcome all these drawbacks, favoring product recovery and improving biocatalyst reusability and stability (Cappa et al. 2016). Non-covalent processes allow the retention of enzymatic activity during immobilization, and are cheap and simple. Immobilization of enzymes via specific domains, such as the poly-His tag, is one of the most popular techniques of enzyme absorption, although the weak binding of enzyme is considered the main disadvantage of this technique (Barbosa et al. 2015). On the other hand, entrapment techniques are also used for biocatalyst stabilization in which cells or recombinant enzymes are enclosed in a porous polymeric matrix to allow the diffusion of substrates and products (Trelles et al. 2004), increasing operational stability, facilitating upstream separation, and bioprocess scale-up.

As is well known, agarose is an economical and chemically inert gelling heteropolysaccharide that possesses an outstanding hydrophilic nature, being ideal for enzyme immobilization (Zucca et al. 2016). Among polysaccharides, sodium alginate is considered an efficient option because it is nontoxic, hydrophilic, biodegradable, and biocompatible. Furthermore, the utilization of nanocomposites such as bentonite in the immobilization procedure is taking greater relevance both as support material for enzyme immobilization and as additive of the polymeric matrix (Rivero et al. 2017; Yeşiloǧlu 2005). Bentonite is a natural nanomaterial that contains a high proportion of swelling clays (smectite) and has a wide range of industrial applications, including catalysts in chemical and oil processing industries, paints, cosmetics, and pharmaceutical technological applications (Holzer et al. 2010).

The present work describes a combination of immobilization techniques such as ionic interaction with high affinity for metal chelates and entrapment in natural matrices improved by bionanocomposites, in order to obtain a stable biocatalyst for the biosynthesis of an anticancer compound known as decitabine, through a green and economical technology.

## Materials and methods

### Materials

Thymidine (dThd), thymine (dThm), and 5-azacytosine (5-azaCyt) were purchased from Sigma Chem. Co. (Brazil). Culture media compounds were obtained from Britania S.A. (Argentina). Chemicals were purchased from Sigma Chem. Co. (Brazil). Sodium alginate was provided by Stanton (Argentina), and Patagonian bentonite was kindly donated by Minarmco Co (Argentina). Agarose 10BCL was purchased from GE-Healthcare (Sweden), and Agarose LE from PB-L (Argentina). HPLC solvents used were supplied by Sintorgan S.A. (Argentina).

### Development of an enzymatic biocatalyst

The enzyme 2′-N-deoxyribosyltransferase from *Lactobacillus animalis* (ATCC 35,046, *La*NDT) was cloned (MN_579049, GenBank accession number), expressed, and immobilized by ionic attachment as was previously described (Méndez et al. 2018). Enzymatic activity of the obtained derivative (IDA-*La*NDT) was evaluated by DAC biosynthesis. The reaction was carried out using dThd (6 mM) and 5-azaCyt (2 mM) in 2-amino-2-hydroxymethyl-1,3-propanediol hydrochloride buffer (Tris–HCl) (20 mM, pH 7) at 30 °C and 200 rpm shaking speed. In such conditions, one unit of enzyme (U) was defined as the amount of enzyme that catalyzed the formation of 1 µmol of DAC in 1 min. Aliquots of 30 µL were taken at different times, centrifuged at 10,000 g, and the supernatant was analyzed by HPLC.

### Derivative nanostabilization

The active derivative IDA-*La*NDT was mixed with 1 mL agarose 3% (w/v) or alginate 4% (w/v), supplemented or not with 0.1% or 0.3% (w/v) bentonite. To obtain the biocatalyst entrapped in agarose, the mixture (derivative-nanocomposite-matrix) was added dropwise to stirred sunflower oil (20 mL) at 10 °C. The resulting gel beads were filtered, washed with hexane and then, with Tris–HCl buffer (20 mM, pH 7), to obtain solvent-free beads. On the other hand, the mixture for alginate entrapment was added dropwise to stirred CaCl_2_ 0.1 M (20 mL) at 25 °C, while the resulting beads were filtered and washed with buffer Tris–HCl (20 mM, pH 7) (Trelles and Rivero 2020). Enzymatic activity of the obtained nanostabilized biocatalysts was evaluated using DAC biosynthesis as previously described.

### Characterization of the nanostabilized biocatalysts

#### Reusability

Operational stability of the biocatalyst entrapped in agarose (*La*NDT-Ag), agarose-bentonite (*La*NDT-AgB), alginate (*La*NDT-Al) and alginate-bentonite (*La*NDT-AlB) was evaluated using DAC biosynthesis as standard reaction. These stabilized biocatalysts were assayed through successive reactions (2 h cycle) until 50% of initial activity loss or matrix integrity loss.

#### Storage stability

Nanostabilized biocatalysts were stored at 4 °C for several days. Then, DAC biosynthesis was evaluated, being defined as the relative activity of DAC conversion between the first and the successive reactions.

#### Thermal stability

To study the thermal stability, the obtained biocatalysts were incubated at 50 °C for different times. Then, the DAC biosynthesis was tested as described previously.

#### Inactivation by solvents

Biocatalyst stability was evaluated after incubation in 40% (v/v) of acetonitrile and dimethylsulfoxide (DMSO) at 25 °C. The relative activity was calculated with respect to the DAC conversion obtained without solvent incubation.

### Bioproduction of DAC

#### Substrate solubilization

In order to solve the low solubility of 5-azaCyt in water and to improve the bioproduction of DAC, the reaction was evaluated by adding the modified base previously dissolved in DMSO. For this, 5-azaCyt solution (0.5 M) in DMSO was added up to solvent final concentration of 2%. This allowed the evaluation of DAC biosynthesis in a 30:10 ratio (dThd:5-azaCyt). It should be noted that substrate concentration and also the addition of 2% DMSO effect on enzymatic activity were evaluated.

#### Scale-up

Batch biotransformation of DAC was evaluated using two systems, packed bed and airlift bioreactor. For the former, a constant flow of 7, 15, 20 and 30 mL/min was evaluated, while an air flow of 0.1, 0.2, 0.4 and 0.6 L/h was assayed for the latter. To maintain a constant reaction temperature (30 °C), a hose through which thermostated fluid circulates was wrapped around the bioreactor. Biosynthesis was carried out using 0.1 g/mL of biocatalyst in optimized reaction conditions: dThd (30 mM) and 5-azaCyt (10 mM) in 15 mL Tris–HCl buffer (20 mM, pH 7).

### Environmental parameters

Green chemistry parameters of the aforementioned bioprocesses were calculated as previously described (Sheldon 2012). Environmental factor (E-factor) is a measure of industrial environmental impact. Carbon efficiency (C-efficiency) and atom economy (A-economy) are parameters designed to evaluate the efficiency of synthetic reactions.

### Analytical methods

Nucleoside biosynthesis reactions were monitored by HPLC (Dionex) equipped with a UV detector (UV/Vis 156, Dionex) using a Nucleosil 10 C18 100A column (10 µm, 300 mm × 4 mm). For DAC biosynthesis an isocratic mobile phase of water/methanol (96:4, v/v) and a flow rate of 1.2 mL/min were used and evaluated at 254 nm; 5-azaCyt, DAC, dThm and dThd retention times were 2.90, 5.66, 7.01 and 16.05 min, respectively.

The obtained product was purified using a Hypersil GOLD column (10 × 150 mm) and an automated fraction collector (AFC-3000, Dionex). DAC purification was carried out at 254 nm using an isocratic mobile phase of water/methanol (96:4, v/v) and a flow rate of 3 mL/min. Retention times of 5-azaCyt, DAC, dThm, and dThd were 3.16, 5.10, 6.83, and 14.43 min, respectively. Afterwards, the purified DAC was concentrated by speed vacuum. Product identification was performed by MS-HPLC using a microtof-Q II BRUKER spectrometer with the electron spray ionization (ESI) method and one ion trap detector.

## Results

### Activity of the enzymatic biocatalyst

**Fig. 1:**
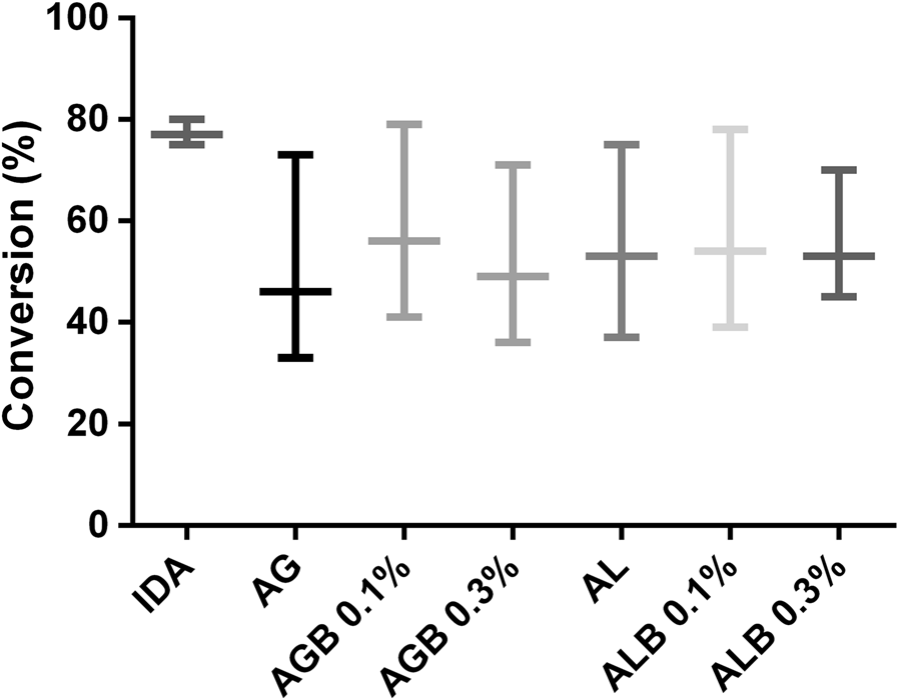
Stabilization effect on enzymatic activity in DAC biosynthesis. The reaction was carried out using dThd (6 mM) and 5-azaCyt (2 mM) in Tris–HCl buffer (20 mM, pH 7) at 30 °C and 200 rpm. Each bar shows the conversion at 1 h (lower end bar) and 5 h (upper end bar). Conversion was calculated as: (mmol product/mmol limiting reagent) × 100. Each letter represents a group with significant differences (p-value < 0.05)

In order to determine IDA-*La*NDT ability to biosynthesize DAC, assays were performed at 30 °C using dThd (6 mM) as sugar donor and 5-azaCyt (2 mM) as modified base. The obtained derivative achieved a conversion of 70% in 15 min of reaction, showing a high productivity (5.6 mM/h). Additionally, the derivative IDA-*La*NDT was able to reach 80% performance after 5 h of reaction (Fig. 1).

### Derivative nanostabilization

With the purpose of obtaining a stabilized biocatalyst easily recoverable, the derivative IDA-*La*NDT was entrapped in agarose and alginate matrix, with or without the incorporation of bentonite. However, the reaction rate during the first hours was affected by the immobilization process. Finally, all the entrapped biocatalysts reached similar conversions to that of the non-entrapped derivative (IDA-*La*NDT) after 5 h of reaction (Fig. 1). Even though there were no marked differences among the entrapped biocatalysts, it can be seen that the one obtained with 0.1% bentonite achieved equal yields to that of the IDA-*La*NDT derivative.

### Characterization of the nanostabilized biocatalysts

#### Reusability

**Fig. 2:**
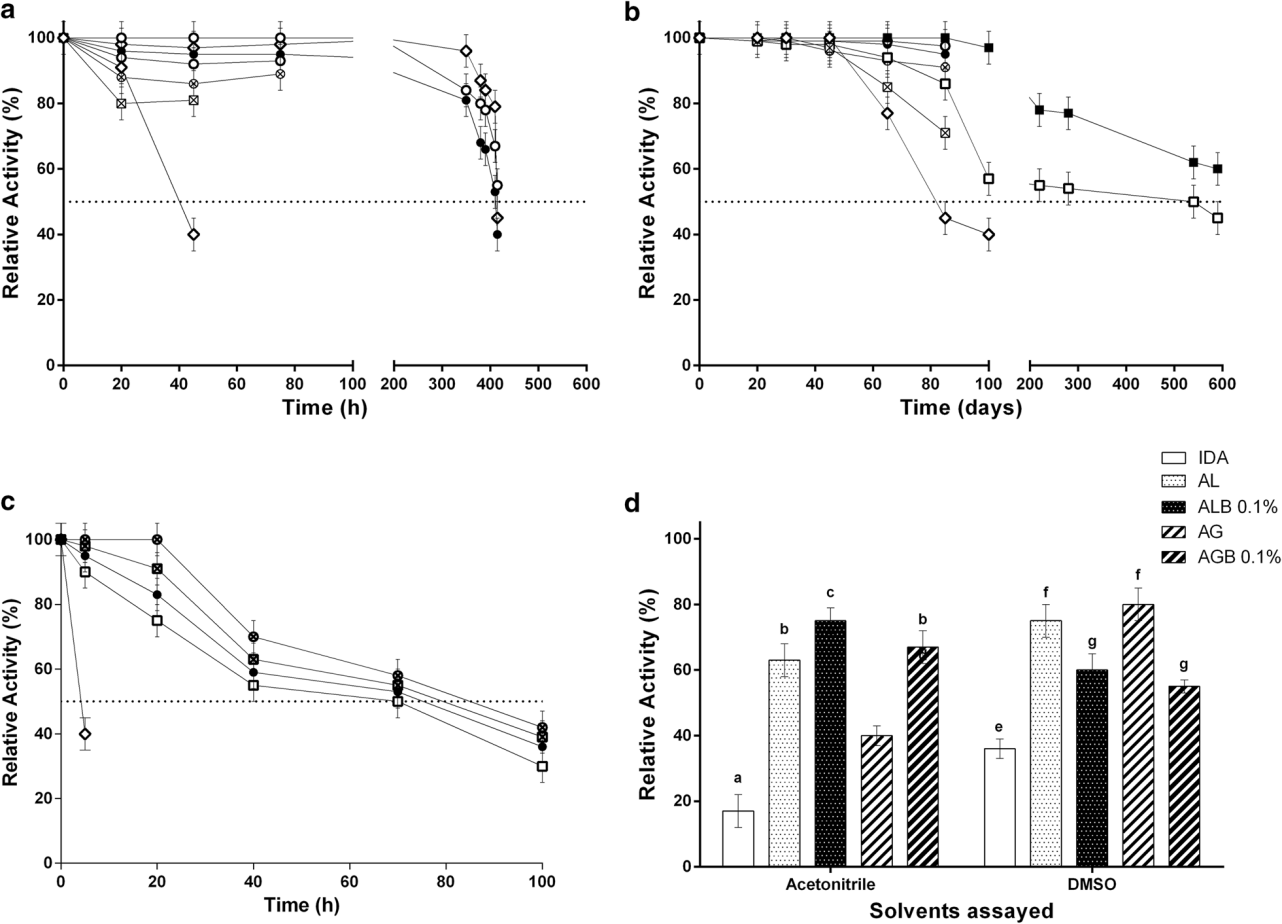
Characterization of the stabilized biocatalysts. **a** Operational stability, **b** storage stability, **c** thermal stability. (▲) Derivative IDA-NDT, (◊) agarose, (□) agarose-bentonite 0.1%, (■) agarose-bentonite 0.3%, (♦) alginate, (○) alginate-bentonite 0.1% and (●) alginate-bentonite 0.3%. **d** Inactivation by solvents. Each letter represents a group with significant differences (p-value < 0.05)

When operational stability was evaluated, the derivative IDA-*La*NDT was inactive in less than 45 h. While the derivatives entrapped in alginate (*La*NDT-Al) and alginate-bentonite (*La*NDT-AlB) maintained their activity for 45 and 75 h respectively, at which time loss of matrix integrity was observed. On the other hand, the biocatalysts stabilized in agarose (*La*NDT-Ag) and agarose-bentonite (*La*NDT-AgB) were significantly more stable than the previous one, maintaining their activity for almost 400 h (Fig. 2a).

#### Storage stability

When storage stability of the obtained biocatalysts was evaluated, it was noted that IDA-*La*NDT biocatalyst was inactive after 77 days, whereas IDA-*La*NDT entrapped in agarose-bentonite 0.1% and 0.3% was active for at least 540 days without activity loss. Nevertheless, biocatalysts entrapped in alginate and alginate-bentonite, as well as that entrapped in agarose, maintained their activity for 85 days (Fig. 2b), until matrix rupture was observed.

#### Thermal stability

Thermal stability at 50 °C was assayed until relative activity of the biocatalyst decreased by more than 50% with respect to the initial one. All the entrapped biocatalysts showed significant improvements in their thermal stability with respect to the derivative IDA-*La*NDT. The stabilized biocatalysts were able to maintain their activity for at least 70 h (Fig. 2c), in contrast to the ionic biocatalyst IDA-*La*NDT, which released the enzyme after 6 h of incubation.

#### Inactivation by solvents

The biocatalysts were incubated in different solvents such as DMSO and acetonitrile (40%, v/v) for 24 h. As a consequence of entrapment, a stabilizing effect was observed, while the activity of IDA-*La*NDT derivative decreased dramatically up to 17% and 36% in acetonitrile and DMSO, respectively (Fig. 2d).

### Bioproduction of DAC

#### Substrate solubilization

As is well known, DAC is slightly soluble in ethanol/water (50:50), methanol/water (50:50), sparingly soluble in water and completely soluble in DMSO. The previous dissolution of 5Aza-Cyt in DMSO allowed performing the DAC biosynthesis reaction with a 30:10 substrate ratio (nucleoside/base). For that, only 2% of co-solvent was necessary, which increased the biocatalytic activity 2.4-fold.

#### Scale-up

**Fig. 3:**
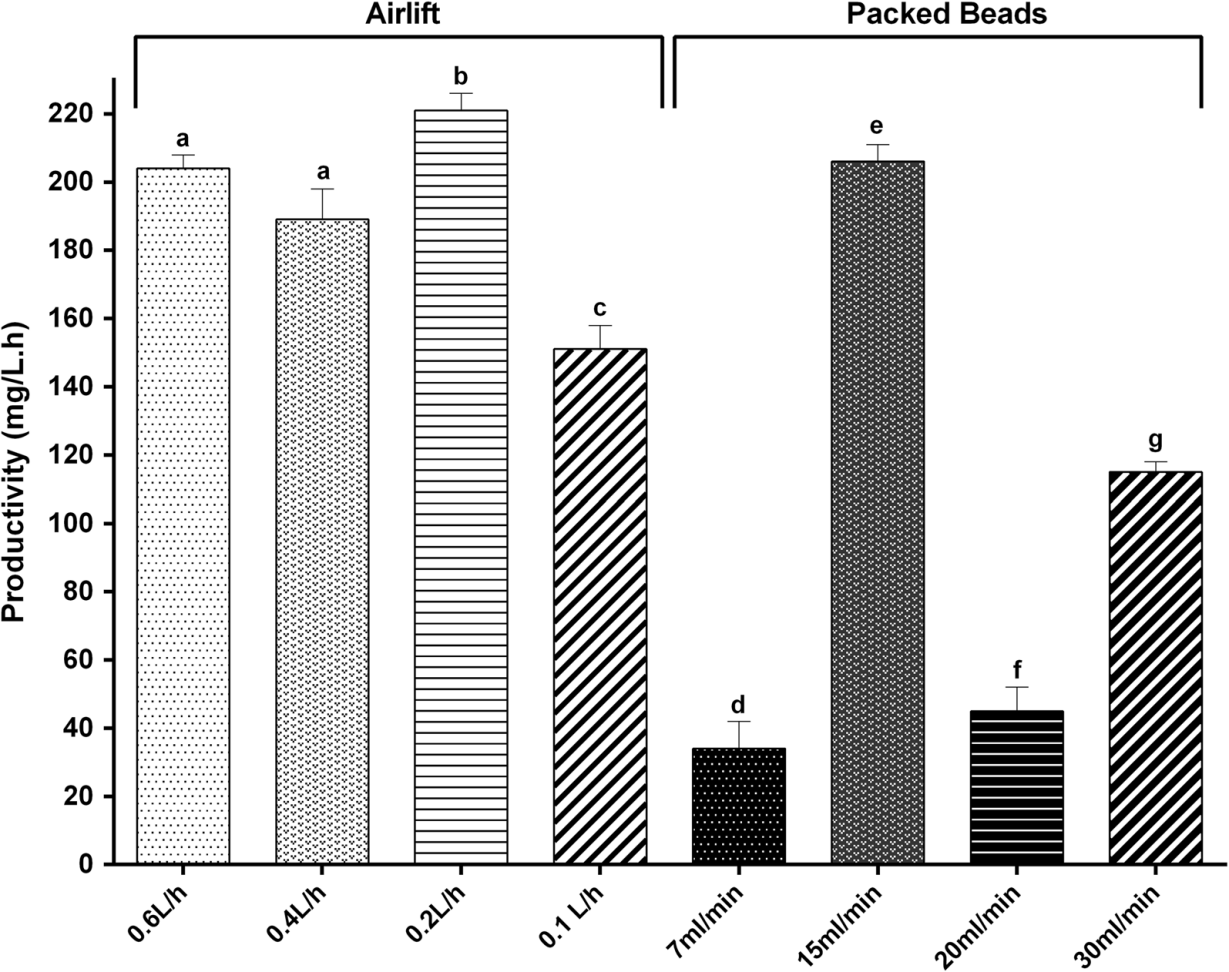
Decitabine scale-up bioprocess. A constant flow of reaction mixture of 7, 15, 20 and 30 mL/min was evaluated in packed bed design, while an air flow of 0.1, 0.2, 0.4 and 0.6 L/h was evaluated in airlift model. Biosynthesis was carried out in 15 mL using 0.1 g/mL of biocatalyst, 30 mM dThd, and 10 mM 5-azaCyt in Tris–HCl buffer (20 mM, pH 7) at 30 °C. The values correspond to 2 h of reaction

Scale-up of the bioprocess using the developed biocatalyst was assayed, an air flow of 0.2 L/h being the best condition for the airlift bioreactor. This kind of bioreactor provides the least mechanical shear to the biocatalyst, increasing its half-life. On the other hand, a flow rate of 15 mL/min was the best condition for the packed bead bioreactor (Fig. 3).

In this sense, bioprocess optimization on a larger scale resulted in maximum productivities of 206 and 221 mg/L h with a packed bed and an airlift bioreactor, respectively. Once the product of interest was obtained in greater amount, its purification was optimized and its subsequent analysis by mass spectrometry with the electron spray ionization (ESI), a mass spectrum was carried out, which allowed the identification of the compound (decitabine; M + : 229.09).

### Environmental parameters

E-factor is a measure of environmental impact generated by industries. E-factor values are around 25–100 for pharmacological compounds. In this work, E-factors less than 6 were obtained for DAC biosynthesis using entrapped biocatalyst *La*NDT-AgB (0.1%).

C-efficiency and A-economy are parameters designed to evaluate the efficiency of chemical synthesis. For DAC biotransformation, these values are shown in Table 1.

**Table 1:** Green parameters for obtaining DAC using the nanostabilized biocatalyst

Green parameters for the biosynthesis of decitabine			
Reaction times	E-factor	C-efficiency (CE)	A-economy (AE)
2 h	5.7	61%	64%
5 h	4.0	61%	64%

## Discussion

First of all, a derivative based on ionic adsorption of *La*NDT (IDA-*La*NDT) was obtained and it was able to yield 70% of DAC conversion in only 15 min, showing a high productivity. Thus a significant improvement in the productivity previously reported by other groups using this kind of enzyme was achieved (Fernández-Lucas et al. 2011; Fresco-Taboada et al. 2016). However, the industrial application of enzymes is feasible only if they are stabilized. So, for the synthesis of DAC, an improved biocatalyst was developed by combining two simple and economical techniques, such as ionic absorption and gel entrapment. When derivative IDA-*La*NDT was entrapped in agarose or alginate, a decrease in DAC biosynthesis rate was evidenced at the beginning of the reaction. However, when the derivative achieved its maximum conversion, after 5 h of reaction, the immobilized biocatalysts reached similar productivities. It is well known that this difference in reaction rate, mainly at the beginning of the reaction, is related to diffusion restrictions of this kind of matrix (Rivero et al. 2012; De Benedetti et al. 2015). However, this slight decrease in the reaction rate is offset by the increase in the number of reuses, in storage stability and in thermal and solvent stability, which was accomplished by the immobilization process. Comparatively, agarose entrapment allowed obtaining the most stable (400 h reuses) biocatalyst with respect to the one obtained with alginate, which showed integrity loss of the matrix after 45 h reuses. This result could be due to the loss of Ca^+2^ ions, which are a fundamental part of the matrix structure (Yong and Mooney 2012).

As is well known, some bionanocomposites such as bentonite have shown improvements in the biocatalyst stability and its mechanical properties when they are incorporated into a matrix such as agarose or a hydrogel such as alginate (Cappa and Trelles 2017; De Benedetti et al. 2015), usually without significantly affecting the biocatalyst activity. Stability improvements of the biocatalyst were evidenced in reusability and storage assays, in which the biocatalysts entrapped in matrices that included bentonite showed the best behavior. The biocatalyst obtained by entrapment in alginate with bentonite showed increased reusability, reaching 75 h of reuses compared to its counterpart without bentonite (45 h), as a consequence of the rigidity conferred by bentonite incorporation (Dos Santos et al. 2015). Although the presence of nanocomposites did not modify the improved reusability provided by the agarose matrix (400 h), the addition of bentonite significantly improved storage stability, staying active for at least 540 h, which is equivalent to a sixfold increase compared to the biocatalyst without incorporation of nanocomposites.

As has been widely reported (Gür et al. 2018; Zucca et al. 2016), hysteresis of agarose and the nature of alginate make them useful reagents in enzymatic entrapment. Since a moderate increase in temperature does not affect the stability of matrices and in order to reduce reaction times of entrapped biocatalysts, DAC biosynthesis was evaluated at 50 °C. Even though a decrease of reaction times for the biosynthesis of DAC (data not shown) was not achieved, the entrapped biocatalysts showed a great stability at this temperature in contrast to the ionic biocatalyst (IDA-*La*NDT), which released enzyme after 6 h of incubation. This greater stability at higher temperatures is consistent with those results reported by Cappa and Trelles (2017). Besides, the entrapped biocatalyst showed improved stability after incubation in 40% of solvents such as acetonitrile and dimethyl sulfoxide with respect to the free biocatalyst. As is well known, in the case of water-miscible organic solvent/water systems, a high organic solvent concentration normally results in suppression of enzyme activity because the organic solvent replaces water molecules on the protein surface layer. Just as was reported by Wan et al. (2010) the greater stability observed in the entrapped biocatalysts could be associated with the microenvironment of the enzyme immobilized on hydrophilic biopolymer, which assists in the retention of the necessary water molecules in the enzyme molecular microenvironment. Moreover, the decrease in enzymatic activity of *La*NDT in such water-organic solvent monophasic systems could be explained by several theories that have been suggested for other enzymes: conformational changes (Öztürk et al. 2010); changes in the active site as a consequence of solvent penetration and differences in substrate solvation (Ryu and Dordick 1992); and perturbation of the quaternary structure (Tóth et al. 2010). Furthermore, DMSO presence in the reaction mixture for DAC bioproduction, even at 2% (v/v), was effective to achieve a 2.4-fold productivity. On the other hand, the scale-up of the bioprocess allowed reaching 221 mg/L.h DAC with an airlift bioreactor. Therefore, considering the reusability of the nanostabilized biocatalyst, it could produce a total of 77 g/L DAC.

Green and sustainable drug manufacturing go hand in hand with forward-looking visions seeking to balance the long-term sustainability of business, society, and the environment (Roschangar et al. 2016). Following this trend, the analysis of the green parameters such as E-factor, related to the total amount of waste generated in the process, C-efficiency and A-economy, theoretical numbers based on a chemical yield of 100%, was carried out. In this work, an E-factor of 4 and 5.7 (2 and 5 h of reaction, respectively), C-efficiency of 64% and A-economy of 61% were obtained, thus showing significantly improved values compared to the ones obtained in the pharmaceutical industry. These results demonstrate mass utilization efficiency and a significant decrease of waste production. Therefore, a smooth, economical and green bioprocess was designed for the production of an antitumoral compound such as DAC. This bioprocess is based on the use of an improved biocatalyst (*La*NDT-AgB 0.1%), which proved to have good efficiency and great stability over time.

## Data Availability

The data supporting the findings can be requested from the corresponding author if necessary.
